# Supplementary figures and images for: The selective culture and enrichment of major rumen bacteria on three distinct anaerobic culture media
Source: Microbiol Spectr. 2025 Sep 30;13(11):e00563-25. doi: 10.1128/spectrum.00563-25 (PMC12584775; doi:10.1128/spectrum.00563-25)

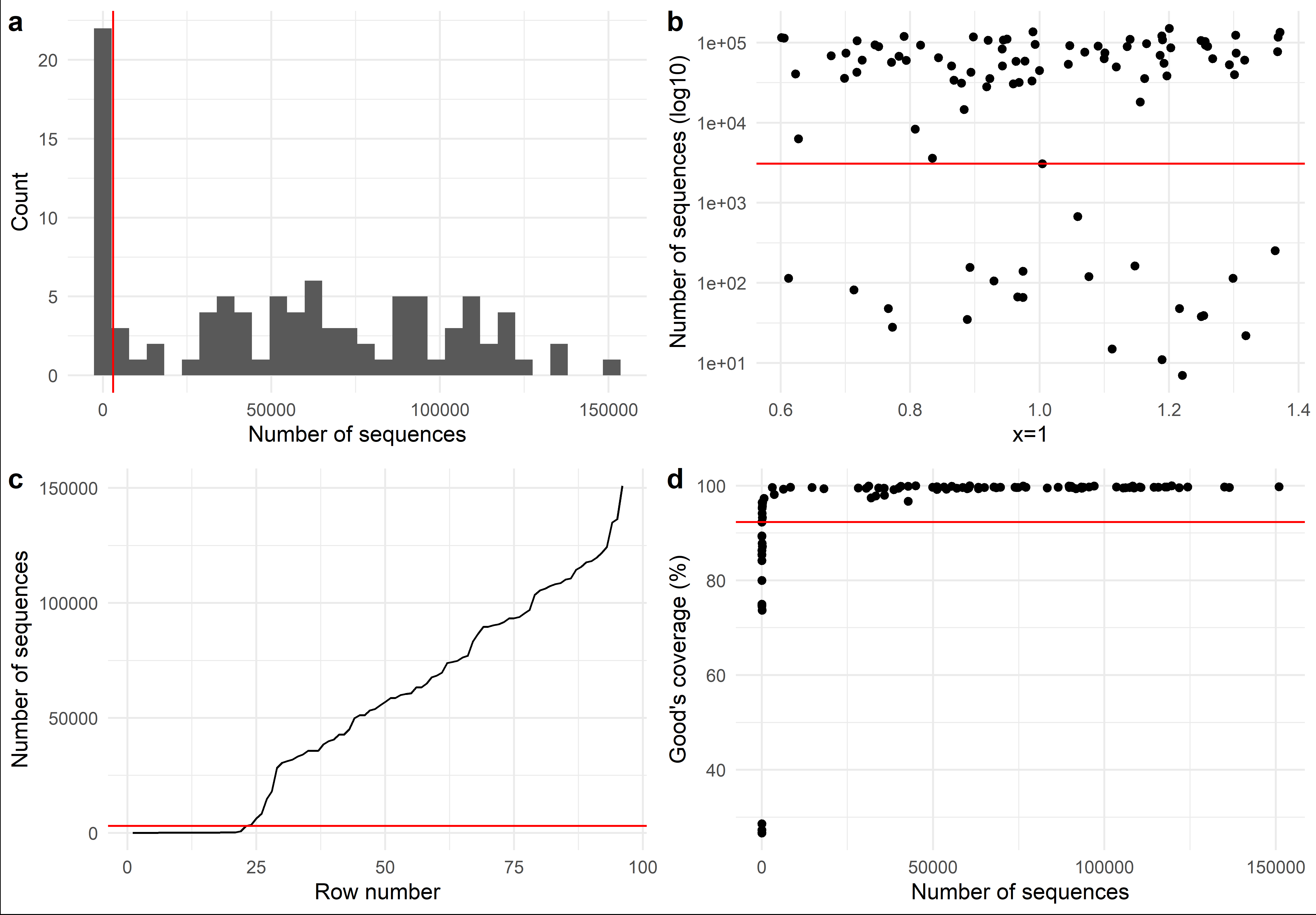

Supplement: Figure S1 — Sequence number cut-off for each sample to be included in the final analysis. [file spectrum.00563-25-s0002.tiff]

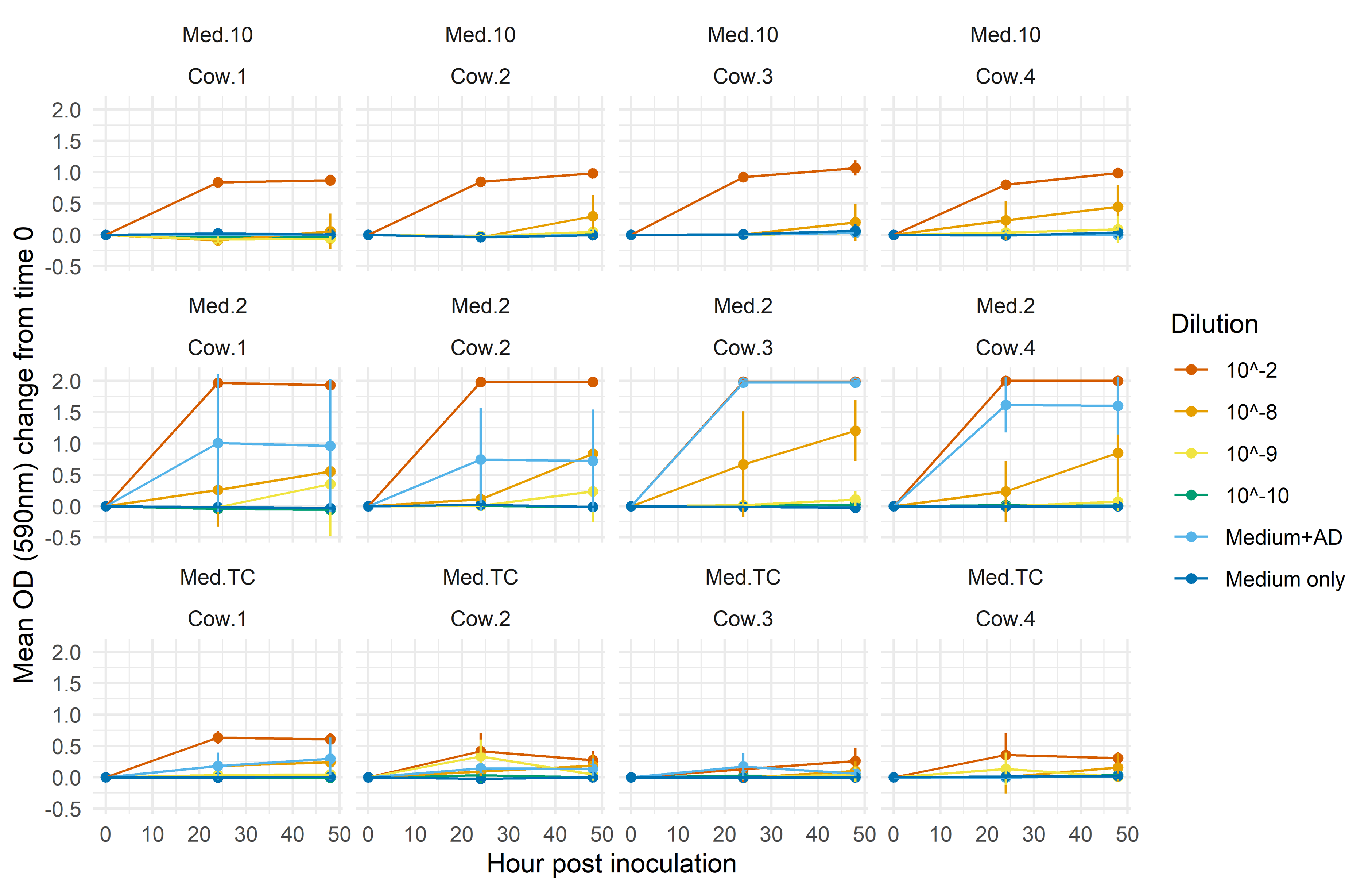

Supplement: Figure S2 — Change in optical density. [file spectrum.00563-25-s0003.tiff]

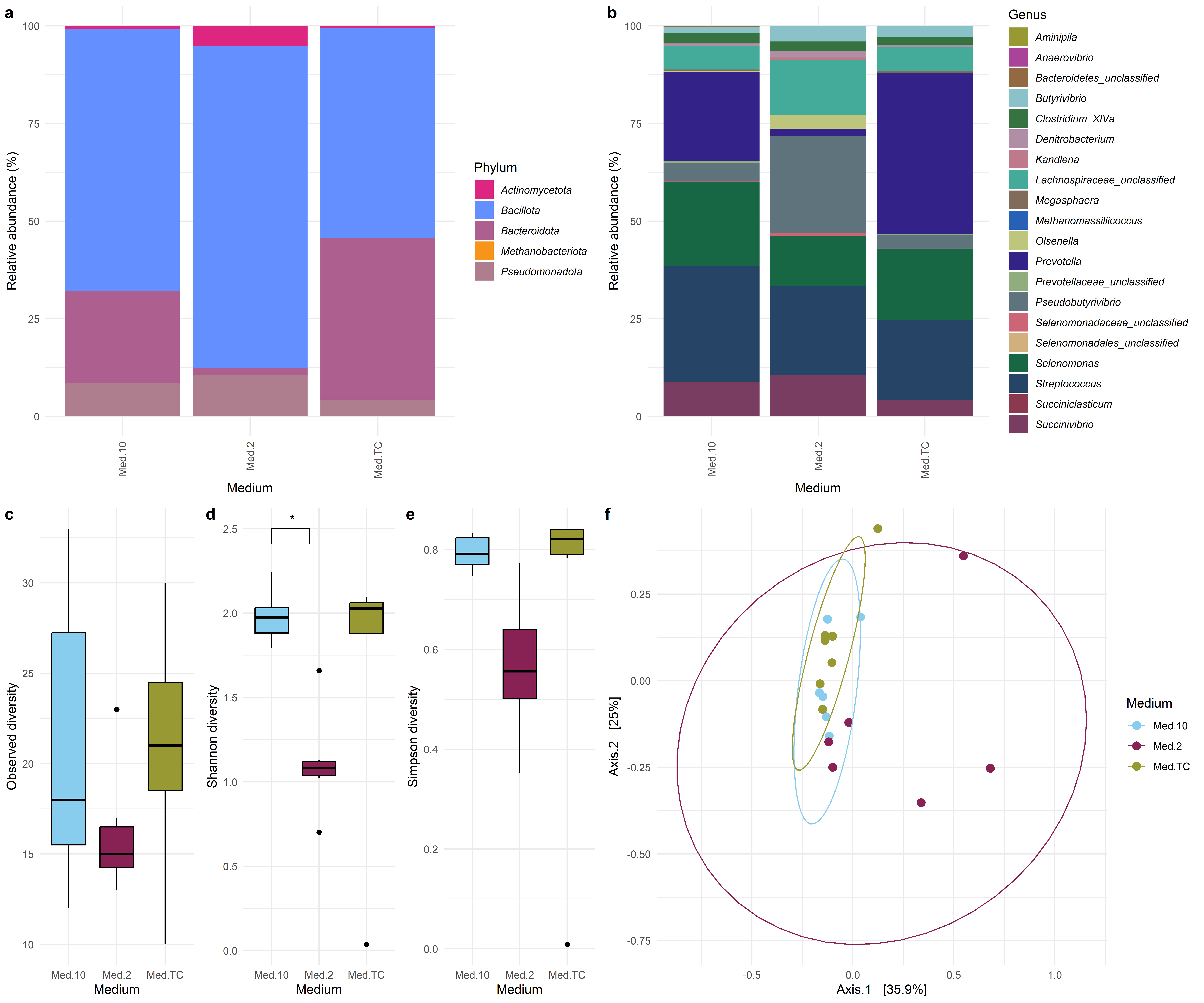

Supplement: Figure S3 — OTUs in the basal media. [file spectrum.00563-25-s0004.tiff]

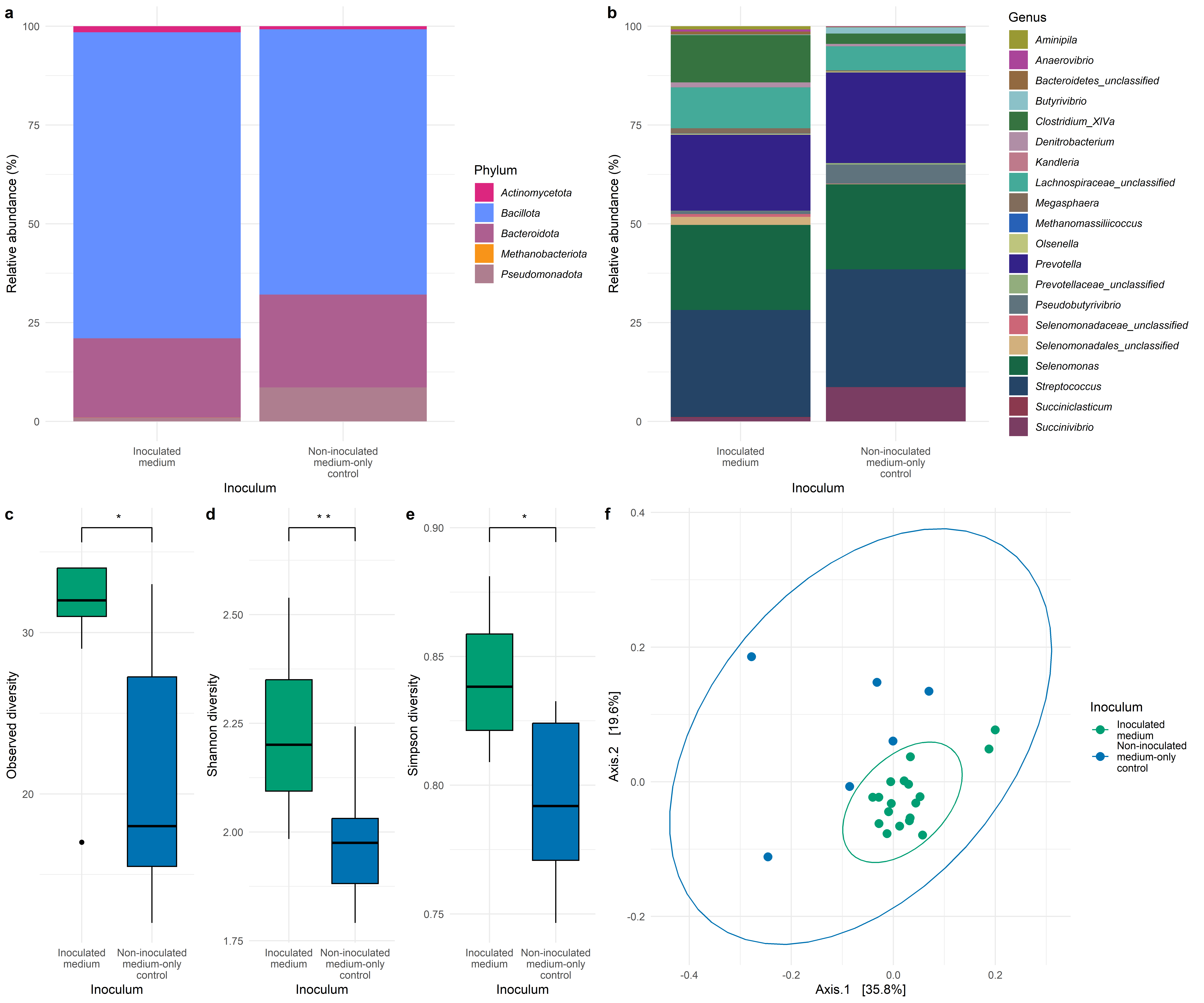

Supplement: Figure S4 — Comparing the OTUs in the Med10 basal medium and those in the inoculated cultures. [file spectrum.00563-25-s0005.tiff]

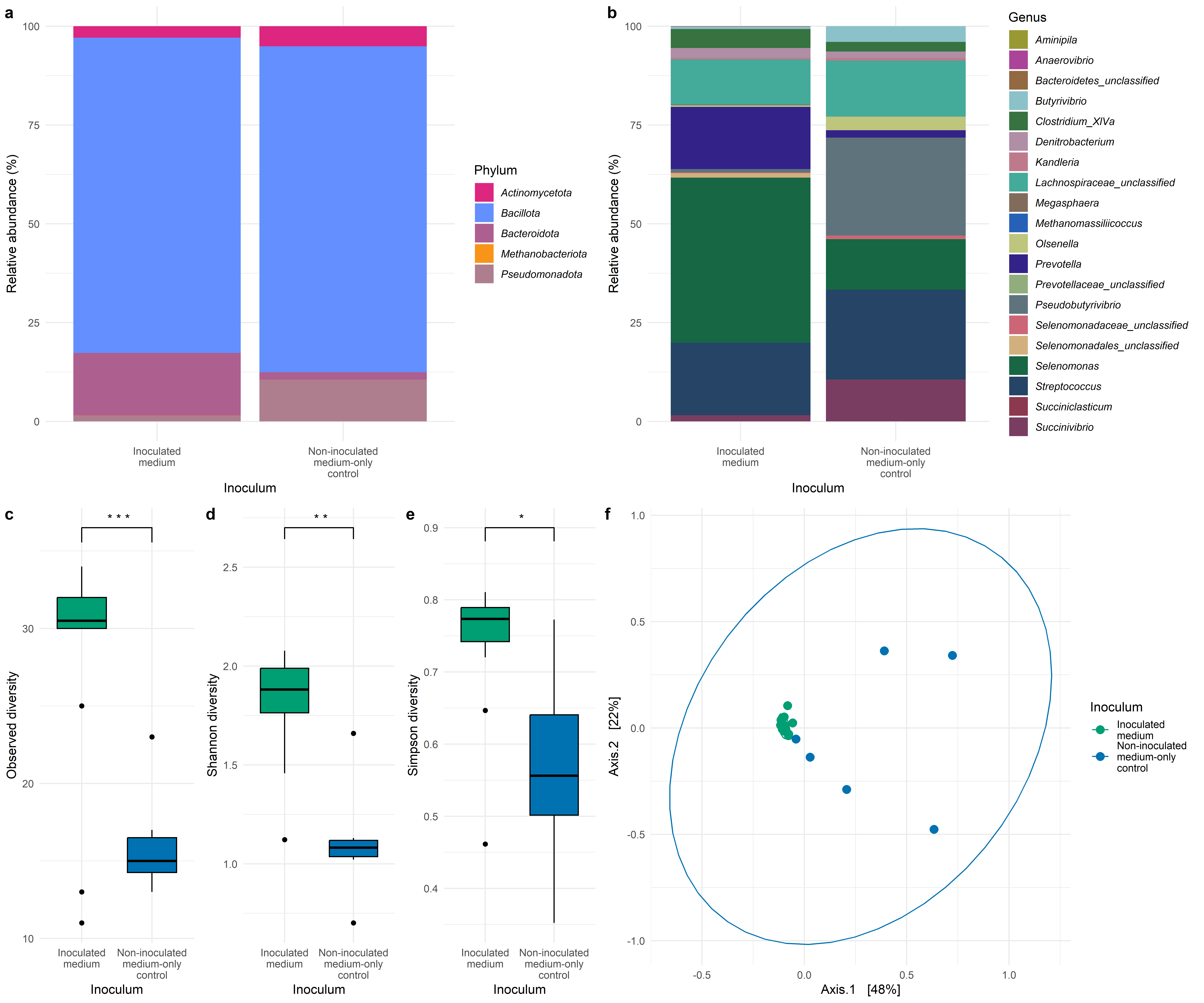

Supplement: Figure S5 — Comparing the OTUs in the Med2 basal medium and those in the inoculated cultures. [file spectrum.00563-25-s0006.tiff]

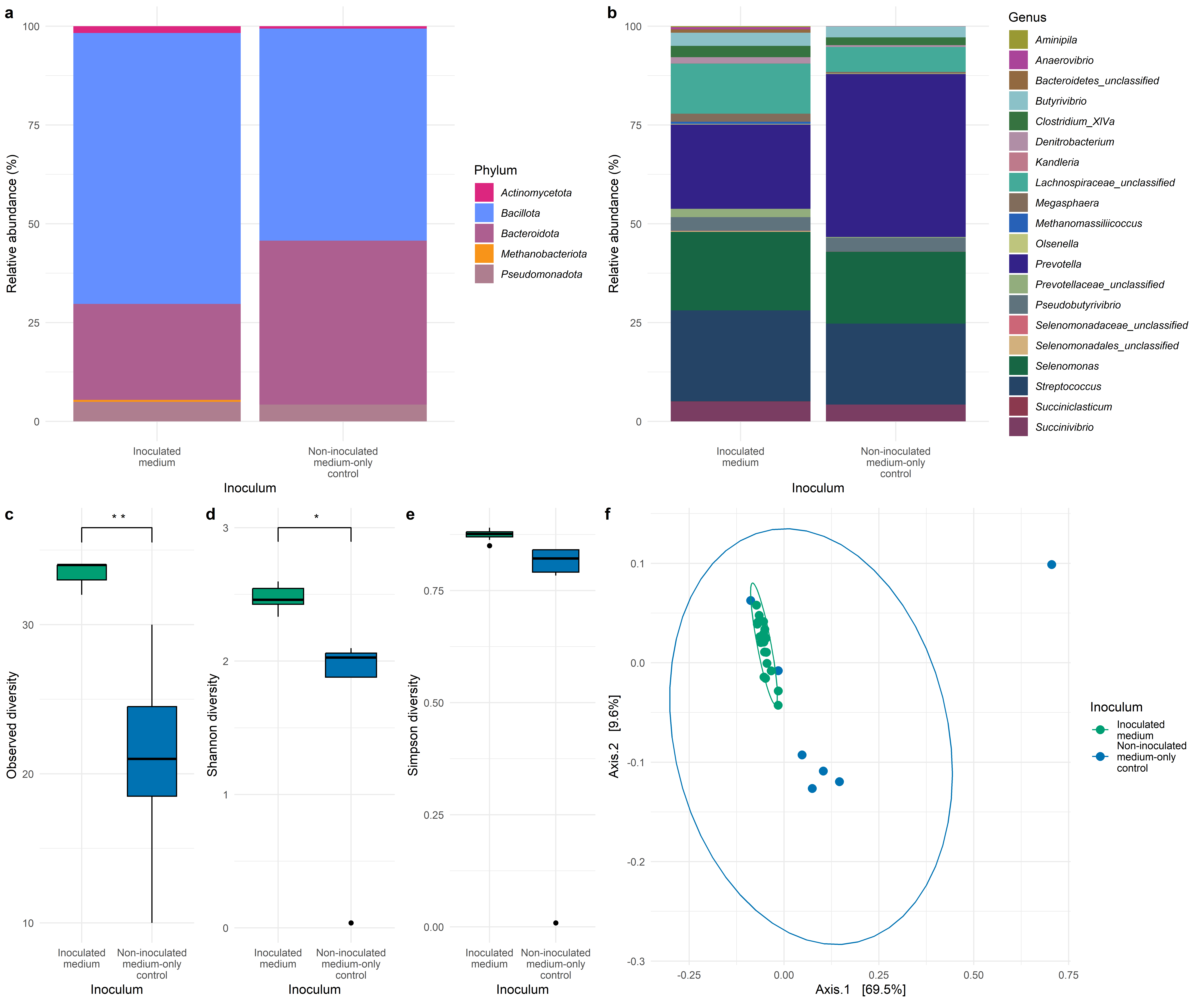

Supplement: Figure S6 — Comparing the OTUs in the MedTC basal medium and those in the inoculated cultures. [file spectrum.00563-25-s0007.tiff]
