# Supplementary material for: The selective culture and enrichment of major rumen bacteria on three distinct anaerobic culture media
Source: Microbiol Spectr. 2025 Sep 30;13(11):e00563-25. doi: 10.1128/spectrum.00563-25 (PMC12584775; doi:10.1128/spectrum.00563-25)
Supplement: Supplemental legends — Legends for Figures S1 to S6 and Tables S1 to S5. [file spectrum.00563-25-s0008.docx]

Figure legends

Figure 1 The relative abundances of phyla (a) and genera (b) found in each replicate culture by cow and medium. Any phyla or genera with a relative abundance < 5% are included in the ‘Other’ category.

Figure 2 Comparison of α-diversity metrics between the microbial communities grown on the three media. a) Observed diversity, b) Shannon diversity, c) Simpson diversity. Statistical tests used are one-way ANOVAs followed by post-hoc Tukey HSD tests if p < 0.05, with the results indicated with P < 0.05 = *, P < 0.01 = **, P < 0.001 = ***.

Figure 3 PCoA plot of weighted UniFrac distances of the cultures highlighted by medium (a) and source cow (b). Statistical tests used are one-way PERMANOVAs followed by post-hoc pairwise-PERMANOVAs with Bonferroni correction tests if p < 0.05, with the results indicated with P < 0.05 = *, P < 0.01 = **, P < 0.001 = ***.

Figure 4 The eight most abundant OTUs (classified to genus level) and their differential relative abundances in the cultured samples by medium. The taxa included are; a) *Selenomonas*, b) *Streptococcus*, c) *Prevotella*, d) *Clostridium* *XIVa*, e) *Prevotella*, f) unclassified *Lachnospiraceae*, g) unclassified *Lachnospiraceae*, h) *Prevotella*. Genera from the *Bacillota* phylum are indicated with ‘Bcl’, and those from the *Bacteroidota* phylum are indicated with ‘Bct’. Statistical tests used are Kruskal-Wallis tests followed by post-hoc pairwise Dunn tests with Bonferroni correction if p < 0.05, with the results indicated with P < 0.05 = *, P < 0.01 = **, P < 0.001 = ***.

Figure 5 The relative abundances of the phyla (a) and genera (b) that make up the microbial communities found in each of the rumen fluid samples from the source cows. All phyla are shown, however any genera with a relative abundance < 5% are included in the ‘Other category.

Figure 6 The mean relative abundances of the phyla (a) and genera (b) that make up the microbial communities found in the cultures grown on each of the types of media compared to the rumen fluid samples, along with (c) the Log2 fold change of the mean relative abundance of each phylum and genus compared to the respective mean relative abundance found in the rumen fluid. Those taxa found in higher mean relative abundances in the rumen fluid are highlighted in red, those in lower mean relative abundances are shown in blue, and those with little change are shown in white.

Supplementary Table 1 ENA metadata linking the ENA codes to sample names and sources.

Supplementary Table 2 An overview of the components of the media and anaerobic diluent, including the composition of each medium, and the composition of the stock solutions used to make the media.

Supplementary Table 3 The 31 OTUs belonging to microbes cultured that were found in a significantly higher relative abundance on one culture medium than another (Kruskal-Wallis rank sum test with Bonferroni correction for multiple comparisons).

Supplementary Table 4 Pairwise comparisons of the microbes that were found in a significantly different relative abundance on at least one medium (post-hoc Dunn test).

Supplementary Table 5 The difference between the relative abundance of each microbe found in the original rumen fluid and it's relative abundance in each of the three media shown through log2 fold change.

Supplementary Figure 1 Change in the optical density (590 nm) of the cultures over time. Some contamination was observed in the Med2 medium and anaerobic diluent control due to the caps being incorrectly sterilised. In this case the OD of the cultures and controls were compared to the medium only controls as a blank. These have been kept in the analysis due to the predicted decrease in the OD of the samples as they were more dilute, the statistically significant difference in diversity between the Med2 cultures and Med2 controls, and the fact the controls were inoculated after the cultures suggesting the contamination happened at the end of the experimental set up rather than at the start when the samples were inoculated.

Supplementary Figure 2 The minimum number of sequences a sample must have to be included in the final analysis. Number of sequencings are plotted against a) sample count, b) x-1, c) row number, and d) Good’s coverage (%). Any samples left of, or below, the red line weren’t included.

**Supplementary Figure 3 A comparison between the OTUs found in each of the basal media.** The mean relative abundance of the phyla (a) and genera (b) are shown, along with differences in the Observed (c), Shannon (d), and Simpson (e) α-diversities, weighted UniFrac beta diversity distances (f). Statistical tests used included one-way ANOVAs followed by post-hoc Tukey HSD tests if p < 0.05 (c-e) and one-way PERMANOVAs followed by post-hoc pairwise-PERMANOVAs with Bonferroni correction tests if p < 0.05 (f). Results are indicated with P < 0.05 = *, P < 0.01 = **, P < 0.001 = ***.

**Supplementary Figure 4 A comparison between the OTUs found in the non-inoculated medium-only controls and inoculated cultures for Med10.** The mean relative abundance of the phyla (a) and genera (b) are shown, along with differences in the Observed (c), Shannon (d), and Simpson (e) α-diversities, weighted Unifrac β-diversity distances (f). Statistical tests used included one-way ANOVAs followed by post-hoc Tukey HSD tests if p < 0.05 (c-e) and one-way PERMANOVAs followed by post-hoc pairwise-PERMANOVAs with Bonferroni correction tests if p < 0.05 (f). Results are indicated with P < 0.05 = *, P < 0.01 = **, P < 0.001 = ***.

**Supplementary Figure 5 A comparison between the OTUs found in the non-inoculated medium-only controls and inoculated cultures for Med2.** The mean relative abundance of the phyla (a) and genera (b) are shown, along with differences in the Observed (c), Shannon (d), and Simpson (e) α-diversities, weighted UniFrac β-diversity distances (f). Statistical tests used included one-way ANOVAs followed by post-hoc Tukey HSD tests if p < 0.05 (c-e) and one-way PERMANOVAs followed by post-hoc pairwise-PERMANOVAs with Bonferroni correction tests if p < 0.05 (f). Results are indicated with P < 0.05 = *, P < 0.01 = **, P < 0.001 = ***.

**Supplementary Figure 6 A comparison between the OTUs found in the non-inoculated medium-only controls and inoculated cultures for MedTC.** The mean relative abundance of the phyla (a) and genera (b) are shown, along with differences in the Observed (c), Shannon (d), and Simpson (e) α-diversities, weighted UniFrac β-diversity distances (f). Statistical tests used included one-way ANOVAs followed by post-hoc Tukey HSD tests if p < 0.05 (c-e) and one-way PERMANOVAs followed by post-hoc pairwise-PERMANOVAs with Bonferroni correction tests if p < 0.05 (f). Results are indicated with P < 0.05 = *, P < 0.01 = **, P < 0.001 = ***.
